# The Role of VCP Mutations in the Spectrum of Amyotrophic Lateral Sclerosis—Frontotemporal Dementia

**DOI:** 10.3389/fneur.2022.841394

**Published:** 2022-02-22

**Authors:** Eveljn Scarian, Giuseppe Fiamingo, Luca Diamanti, Ilaria Palmieri, Stella Gagliardi, Orietta Pansarasa

**Affiliations:** ^1^Department of Brain and Behavioral Sciences, University of Pavia, Pavia, Italy; ^2^Cellular Models and Neuroepigenetics Unit, IRCCS Mondino Foundation, Pavia, Italy; ^3^Neuroncology Unit, IRCCS Mondino Foundation, Pavia, Italy; ^4^Department of Molecular Medicine, University of Pavia, Pavia, Italy; ^5^Neurogenetics Research Center, IRCCS Mondino Foundation, Pavia, Italy; ^6^Molecular Biology and Transcriptomics Unit, IRCCS Mondino Foundation, Pavia, Italy

**Keywords:** ALS, FTD, VCP, protein clearance, autophagy, lysophagy, mitophagy

## Abstract

Amyotrophic Lateral Sclerosis (ALS) and Frontotemporal Dementia (FTD) are two neurological diseases which, respectively, and primarily affect motor neurons and frontotemporal lobes. Although they can lead to different signs and symptoms, it is now evident that these two pathologies form a continuum and that hallmarks of both diseases can be present within the same person in the so-called ALS-FTD spectrum. Many studies have focused on the genetic overlap of these pathologies and it is now clear that different genes, such as *C9orf72, TARDBP, SQSTM1, FUS*, and *p97/VCP* can be mutated in both the diseases. *VCP* was one of the first genes associated with both FTD and ALS representing an early example of gene overlapping. VCP belongs to the type II AAA (ATPases Associated with diverse cellular activities) family and is involved in ubiquitinated proteins degradation, autophagy, lysosomal clearance and mitochondrial quality control. Since its numerous roles, mutations in this gene lead to different pathological features, first and foremost TDP-43 mislocalization. This review aims to outline recent findings on *VCP* roles and on how its mutations are linked to the neuropathology of ALS and FTD.

## Introduction

Amyotrophic Lateral Sclerosis (ALS) and Frontotemporal Dementia (FTD) are two neurodegenerative diseases which affect motor neurons and frontotemporal lobes, respectively. Traditionally ALS was considered as limited to the motor neuron system; in the last decades a wide spectrum of possible cognitive and behavioral deficits, similar to the ones mostly seen in the behavioral variant of FTD (bvFTD), have been highlighted. Further evidence of the overlap between the syndromes has become evident through genetic, pathological, radiological and neuropsychological studies (1). It is now evident that the two diseases represent the opposite poles of a phenotypic continuum. The mixed phenotypes, constituted by a variable burden of motor and extra-motor deficits, are probably as frequent as the pure forms. The possible phenotypes related to ALS and FTD are still expanding due to the association of rarer genes already known to be causative of other systemic diseases (2, 3). One such example is Valosin Containing Protein (*VCP*), on which this review will be focused.

## Clinical Aspects of ALS and FTD

ALS is a rare neurodegenerative disease primarily affecting the corticospinal tract and leading to progressive muscular paresis. Its peak age of onset is between 50 and 70 years and it is characterized by the simultaneous presence of symptoms and signs of both upper (UMN) and lower motor neuron (LMN). Common histopathological features are transactivation response DNA-binding protein 43 kDA (*TDP-43*)-positive cytoplasmic inclusions and neuronal degeneration with variable distribution and spreading (4). The diagnosis, according to El Escorial Criteria [first established in 1994 (5) and later revised (6, 7)] is clinical and supported by needle electromyography (EMG). As regards treatment, a few pharmacological options are available with only scant efficacy in slowing down the neurodegenerative process (8). An upcoming promising therapeutic approach is targeted-gene silencing through antisense oligonucleotide drugs intrathecally delivered (9).

FTD is the third leading cause of dementia and the second most common early-onset dementia after Alzheimer's Disease (AD). FTD encompasses different clinical and neuropathological subtypes according to consensus criteria (10–12). The most frequent FTD syndrome is bvFTD (nearly 70%), characterized by personality changes, dishinibition, apathy, hyperorality and executive dysfunction. Less represented language predominant variants are Semantic Dementia (SD) and Progressive Non-Fluent Agrammatic variant (PNFA) (13, 14). A third primary progressive aphasia (PPA) syndrome, the logopenic variant (lvPPA), is mainly attributed to AD pathology and the beta-amyloidogenic pathway (15). In bvFTD, the neurodegenerative process is predominantly focused on the prefrontal cortex, while temporal lobes are involved later on. On the contrary, PPA neurodegeneration seems to start primarily in the temporal lobes, with asymmetrical pattern (16, 17). Nearly all cases of frontotemporal lobar degeneration (FTLD) are driven by microtubule-associated protein tau (MAPT), TDP-43 or the fused-in-sarcoma (FUS) protein accumulation (18). FTD lacks disease-modifying therapies; behavioral symptoms can benefit from antidepressant and antipsychotic agents (19).

### Phenotypic Variability of ALS and ALS-FTD Overlap

The clinicopathological variability of ALS is remarkable. The classical depiction of UMN and LMN concomitant involvement (Charcot's disease) is seldom present at the onset of disease; this motor variability reflects the site of onset of the neurodegenerative process, its rate of progression and subsequent spreading to different neuroanatomic areas. This heterogeneity actually represents a continuum that makes patients classification troublesome. However, some phenotypes are widely accepted because of reproducible clear-cut features (20). The predominance of LMN system is called progressive muscular atrophy (PMA) (21), while the predominance of UMN system configures primary lateral sclerosis (PLS) (22). According to the predominant muscle territory involved or the site of onset, ALS can manifest a bulbar or a spinal onset. A respiratory phenotype, with diaphragmatic failure since the onset of the disease, is rarely seen (23). ALS phenotypic differentiation is more easily accounted at disease onset. Later on, motor neuron degeneration spreads in adjacent segments or along the corticospinal tract (24). Population-based studies have highlighted different epidemiological distributions in the above-mentioned phenotypes, according to sex, age, prognostic value, and cognitive profile (20, 25).

Concerning neuropsychological (NPS) impairment, first reports of ALS patients with frontal lobe deficits or dementia date back to the nineties (26, 27). Already then, the cognitive and behavioral profile in ALS population was described as similar to the ones typical for bvFTD. Further evidence of ALS-FTD overlap came from the observation of motor deficits and/or signs in FTD patient (28) and the identification of common TDP-43 neuropathologic inclusions in both syndromes (29). The discovery of a shared FTD and ALS causative gene mutation, *C9orf72* hexanucleotide GGGGCC (G4C2) repeat expansion, definitely confirmed previous hypotheses of a common pathogenic pathway (30). Overall, ALS is most typically associated with bvFTD, and the ALS-FTD overlap syndrome shows the strongest association with TDP-associated FTLD (FTLD-TDP) (18).

The ever-growing evidence of an ALS-FTD disease-spectrum led to operational consensus criteria aimed at classifying ALS according to motor features as well as extra-motor NPS features (31, 32). Through standardized NPS assessment, different shades of cognitive and behavioral impairment are categorized as follow: ALS-behavioral impairment (ALSbi); ALS-cognitive impairment (ALSci), with typical either executive and/or language dysfunction; ALS-cognitive and behavioral impairment (ALScbi), when meeting both criteria for ALSci and ALSbi (33); ALS-FTD, when ALS patients also show the presence of bvFTD or PPA, according to the respective diagnostic criteria (10, 12). Generally speaking, cognitive and/or behavioral impairment occurs in up to 50% of ALS patients, while nearly 15% have a full-blown bvFTD (34–36). Taking into account dementia-first patients, almost 15% of bvFTD patients develop ALS during the course of the disease, while signs of motor neuron impairment are observed in about 40% of cases (37, 38).

Both cognitive and behavioral impairment, with special consideration to apathy, negatively correlate with survival (39). A recent study shows that ALS-FTD patients' survival is significantly shorter than pure bvFTD patients'; in addition, among ALS-FTD patients, motor-onset patients' survival is much shorter than cognitive-onset ones, meaning that disease progression is accelerated when motor deficit come first (40). To better elucidate the reciprocal interplay between ALS and FTD, genetics has progressively shed some light.

## Genetics of ALS, FTD, ALS + FTD

From the discovery of the first missense mutations in superoxide dismutase 1 (*SOD1*) in 1993 (41) to our date, over 50 genetic mutations have been linked to ALS (3), thanks to genome-wide association studies and “next-generation” sequencing techniques (42). Yet, these advancements have allowed to better explain only a minor fraction of cases so far. About 10% of ALS patients are considered familiar (fALS), as they have at least one other affected family member. However, one-third of fALS cases remain genetically unexplained (43). Considering the remaining 90–95% sporadic ALS (sALS), just about 10% carry a mutated causative gene, accordingly to current knowledge. This missing heritability in ALS may be due to technical issues as well as the inherent complexity of the disease (3).

Four genes, *SOD1, C9orf72, FUS*, and *TARDBP*, account for the majority of both fALS and sALS cases (44) (Table 1).

**Table 1:** List of genes involved in ALS and FTD pathologies.

**Gene**	**Locus**	**Mutations**	**First description of the gene**
*ANG* (Angiogenin)	14q11.2	86	Fett et al. (45)
*ATXN2* (Ataxin 2)	12q24.12	60	Gispert et al. (46)
*C9orf72* (chromosome 9 open reading frame 72)	9p21.2	161	DeJesus- Hernandez et al. (30)
*CHCHD10* (Coiled-Coil-Helix-Coiled-Coil-Helix Domain Containing 10)	2*2q1*1.23	205	Bannwarth et al. (47)
*CHMP2B* (Charged Multivesicular Body Protein 2B)	3p11.2	110	Babst et al. (48) (Vps2 hortolog of CHMP2B)
*DCTN1* (Dynactin Subunit 1)	2p13.1	565	Holzbaur et al. (49)
*FUS (FUS RNA binding protein)*	1*6p1*1.2	323	Crozat et al. (50)
*GRN (Granulin Precursor)*	1*7q2*1.31	302	Zhou et al. (51)
*MAPT (microtubule associated protein tau)*	1*7q2*1.31	465	Goedert et al. (52)
*MAT*R3* (Matrin 3)*	*5q3*1.2	249	Belgrader et al. (53)
*NE*K1** (NIMA Related *Kinase 1)*	*4q3*3	418	Letwin et al. (54)
*OPTN (Optineurin)*	1*0p1*3	240	Li et al. (55)
*PF*N1** (Profilin 1)	1*7p1*3.2	70	Carlsson et al. (56)
*SETX (Senataxin)*	*9q3*4.13	941	Moreira et al. (57)
*SO*D1**(Superoxide *dismutase 1)*	2*1q2*2.11	208	Rosen et al. (41)
*SP*G1*1 (Spatacsin)*	1*5q2*1.1	*1494*	Stevanin et al. (58)
*SQST*M1* (Sequestosome 1)*	*5q3*5.3	383	Park et al. (59)
*TARDBP (TAR DNA Binding Protein)*	*1p3*6.22	214	Ou et al. (60)
*TB*K1* (TANK Binding Kinase 1)*	1*2q1*4.2	206	Pomerantz and Baltimore (61)
*VCP (valosin containing protein)*	*9p1*3.3	356	Koller and Brownstein (62)

*Genes are reported with the corresponding locus, numbers of known mutations and reference of the first description*.

*SOD1* encodes a superoxide dismutase enzyme which resides in the cytosol and the intermembrane space of mitochondria. This enzyme catalyzes the production of oxygen and hydrogen peroxide from the superoxide species produced during cellular respiration, providing an antioxidant mechanism (63). *SOD1* mutations are considered responsible for 15–30% of fALS and fewer than 2% of sALS cases (64). To date, 217 disease-associated variations in *SOD1* have been identified,^1^ they are usually missense and lead to a decrease in enzyme activity and related oxidative stress and mitochondrial dysfunction (65). The pathology of *SOD1* ALS seems distinct from that of all other types of ALS, in that it lacks the TDP-43 and/or FUS pathology hallmarks (66).

The first genetic link between ALS and FTD was the discovery of mutations in *TARDBP*, which encodes TDP-43 (67). TDP-43 is a DNA/RNA binding protein which shuttles back and forth between the nucleus and cytoplasm (68) and regulates gene expression and RNA processing (69).

To date, 69 variants in *TARDBP* have been linked to ALS,^2^ and they can variably lead to the loss or the overexpression of TDP-43, both causing disease. Indeed, TDP-43 homeostasis is critical for cell survival and it is tightly regulated: when excessive, it can form inclusion bodies in the cytoplasm; when depleted, it may result in mRNA metabolism dysregulation (70). Furtherly, a prion-like spreading mechanism has been shown for TDP-43 mutated products (71). Despite a central role in ALS pathogenesis, the frequency of the gene mutation remains much lower than the occurrence of TDP-43 neuropathologic neuronal inclusions (about 4% of fALS) (3).

Shortly after *TARDBP*, missense mutations of *FUS* were identified (72). FUS protein shares functional homology with TDP-43, playing a role in RNA metabolism and nucleocytoplasmatic transport (73). FUS is also involved in DNA repair (74) and paraspeckles formation against stressful noxa (75). One hundred twenty-six autosomal dominant *FUS* mutations are known to cause FUS cytoplasmatic aggregation,^3^ which occurs almost exclusively in association with *FUS* pathogenic variants (76, 77), while TDP-43 aggregation is seldom present in *FUS*-ALS patients.

As already mentioned, the major contribution in ALS-FTD genetic linkage has been the discovery of the hexanucleotide repeat expansion (GGGGCC) in the non-coding region of the *C9orf72* gene (30). *C9orf72* mutation is the most common inherited cause of fALS (approximately 34%) worldwide and in the European population (64). The exact C9orf72 function is still poorly understood, as is its broad phenotypic expression. Endosomal trafficking, autophagy (78) and immune dysregulation (79) are proposed functions. *C9orf72* expansion, seems to confer a loss of function (80). Toxic gain of function is also theorized, since the expanded transcript forms stable secondary structures unable to effectively interact with other proteins, leading ultimately to impair RNA processing (81).

Next-generation sequencing has allowed the identification of numerous rarer genetic variants in many other genes related to ALS, whose function has been linked to RNA processing, protein homeostasis, cytoskeletal dynamic, mitochondrial function, or still unknown (Table 1). Although rare, their discovery is contributing to better understanding ALS pathogenic mechanisms (3, 82).

As regards FTD genetics, familiar hereditability and mutation rates are high in comparison to ALS, with great variability across the clinical phenotypes. Almost 50% of bvFTD patients have a strong family history; conversely, PPA occurs in a familiar fashion only in 12% of people (83). *C9orf72*, as for ALS, is the commonest cause of genetic FTD worldwide (about 25%), followed by *GRN* (10–20%) and *MAPT* (10–20%) (2). These three genes cover the vast majority of familiar FTD cases and are inherited in an autosomal dominant fashion (Table 1).

*MAPT* is the first gene associated with FTD, whose main function is microtubule stabilization. Mutations in protein tau result in hyperphosphorylated tau deposits, which are toxic for cell homeostasis without amyloid pathology, as seen in AD. To date, over 50 pathogenic mutations are known (84). *MAPT* accounts for 10–20% of familiar FTD and 0–3% of sporadic FTD (85).

GRN is a glycoprotein mainly expressed in myeloid cells and a subset of neurons, especially cerebral cortical neurons, motor neurons, Purkinye and hippocampal cells (86). It plays an important role in inflammation modulation, tissue repair and neuronal survival (87). *GRN* mutations are heterozygous and result in 75% loss of *GRN* (88). They are responsible for 5–20% of familial and 1–5% of sporadic FTD cases. *GRN*-related FTD is characterized by TDP43 proteinopathy, although it is not clear how *GRN* mutation impairs *TARDBP43* metabolism.

Multiple other FTD-linked genes are known, although cumulatively accounting for <5% of cases (2). Among such genes, *VCP, CHMP2B, TARDBP, FUS, SQSTM1, ANG, CHCHD10, TBK1, OPTN, NEK1* have also been described in ALS patients (2), providing further evidence of a genetic overlap in ALS-FTD clinical continuum.

## Genotype-Phenotype Correlations

### SOD1

Over 185 *SOD1* disease-associated variations have been identified. Some of them have been linked to faster disease progression (A4V, H43R, L84V, G85R N86S, and G93A), longer life expectancy (G93C, D90A, or H46R) (89) and specific genotype-phenotype correlations. A4V variant determines a relentless fast limb-onset disease with an average of 1 year survival (90). D90 variant acts differently in relation to is mutational state: if homozygous, the disease is generally slow, with survival up to 14 years, and ascending from inferior to superior limbs (91); if heterozygous, the disease is more severe, with bulbar or upper limb onset and faster progression (92). Overall, *SOD1* typically confers more LMN than UMN involvement, and cognitive impairment is not generally reported in *SOD1* disease.

### TARDBP

*TARDBP* patients have an early onset with upper limb predominance and longer disease-duration compared to sALS, non-mutated fALS and *SOD1*+ ALS (93). Mutational state can strikingly affect survival, from 27 months with G298S to over than 100 in M337 carriers (93). *TARDBP* is seldom associated with cognitive impairment or full-blown FTD (94). When cognition is affected, language deficits and temporal atrophic changes on imaging are frequently seen (95).

### FUS

*FUS* variants are associated with early onset and juvenile ALS (96), and *FUS* genotype correlates with faster disease progression in comparison with *SOD1*+ and *TARDBP*+ patients (97). Dormann et al. (98) demonstrated that point mutations (R521G, R522G, R524S, P525L) in the FUS C-terminal domain impair nuclear import and enhance FUS cytosolic accumulation at a varying degree according to the mutation. This finding is in accordance with the evidence of shorter survival for fALS patients with P525L and R522G mutations, which determine the highest degree of FUS cytosolic inclusions (72, 99). As for *TARDBP, FUS* does not express prominent FTD overlap (94); remarkably, FTD patients with FUS pathology never carry FUS or other known mutations, lack motor or language involvement and manifest a predominant obsessive-repetitive behavioral impairment (100).

### C9orf72

The whole phenotypic ALS-FTD spectrum has been linked to *C9orf72* expansions. ALS motor phenotypes can be all expressed in *C9orf72* carriers, with a slight higher incidence of bulbar onset compared to other fALS (101). Among FTD variants, bvFTD with executive dysfunction is the most frequent, though language-predominant phenotypes meeting SD and PNFA are also possible. Median survival in C9orf72-carriers is shorter than non-carriers (102). In a French study the disease duration of C9orf72-related ALS was significantly shorter than in patients with mutations in *SOD1, TARDBP* or other familial ALS cases, while disease onset was significantly later in *C9orf72*-related ALS compared to *SOD1* and *FUS*-ALS (101).

*C9orf72* expansion carriers may have an atypical neuropsychiatric presentation with associated hallucinations or delusions and a greater risk of psychiatric disorders (103).

Finally, patients co-expressing *ATXN2* intermediate repeats are more likely to have a pure form of ALS. The striking variability in the phenotype associated with the *C9orf72* expansion suggests that multiple modifiers may exist, either genetic and/or environmental. The other two main FTD-associated genes, *GRN* and *MAPT*, are never associated with motor features suggesting an ALS-FTD overlap (104).

The fourth most frequent FTD-associated gene, *TBK1*, has been linked to prominent PPA features as well as isolated ALS and ALS-FTD (105).

## Phenotypic Pleiotropy: The Case of VCP

Other genetic diseases, affecting organs and systems seemingly unrelated to the nervous system, have been linked to ALS-FTD continuum (3). This is the case of genes of minor frequency and involvement such as *OPTN, SQSTM1*, and *VCP*, which show remarkable phenotypic pleiotropy.

Mutations in *VCP* were already known to underlie an unusual clinical syndrome characterized by inclusion body myopathy, Paget's disease of the bone and FTD (IBMPFD), or multisystem proteinopathy (106). In IBMPFD, a myopathic pattern with progressive weakness and atrophy of proximal skeletal muscles is the most common feature (90%). Histologically, rimmed vacuoles and TDP-43 positive inclusion are seen in the involved muscles (107).

*VCP*-associated Paget's disease typically involves the spine, pelvis, scapulae and skull, leading to their structural disruption by bone remodeling blockage. *VCP*-related FTD is characterized by TDP-43 inclusions (108). The relative low frequency of FTD (nearly 30%) in IBMPFD is thought to be a consequence of the early age at death in most patients. When present, FTD reduces life span to an average of 6 years (109).

Mutations in *VCP* have also been associated with pure ALS (110), hereditary spastic paraplegia (111), Charcot-Marie-Tooth type 2 disease (112), and multiple dystrophic syndromes (113). Hence, *VCP* mutations result in pathology on both sides of the neuromuscular junction and show that extra-neurologic tissues, such as muscle and bone, may be affected. Most cases of IBMPFD and less typical VCP-related diseases are caused by heterozygous missense mutations (106).

Mehta et al. (114) studied genotypic-phenotypic relations in 27 families (190 subjects, 45 carriers and 145 symptomatic) by grouping them according to *VCP* variants: 91% of patients had myopathy, 51.7% showed Paget's disease and 30.3% had FTD. R155C mutation was associated with shorter survival, while intergroup analysis was limited by the small sample size of each group. ALS was reported in 13 subjects (8.9%) from 6 different families, 10 of them carrying R155H mutation; furthermore, 24% of the symptomatic subjects had neurogenic changes on the EMG (114). The coexistence of myopathic and neurogenic findings has been reported by other authors, as well (115). Similar results for phenotypic distribution were later confirmed by the same group of authors (116), who also reported autopsy data on one individual with *VCP*-related ALS showing typical Bunina bodies and TDP-43 immunostaining within nervous tissue. In their cohort, the classic IBMPFD triad of symptoms was manifest in only 10% of cases (116). A very small percentage of cases was diagnosed as Parkinson's disease or AD. *VCP*-related ALS and FTD are phenotypically indistinguishable from sporadic forms. The only remarkable feature of *VCP*-attributed FTD is a younger onset (114). Although rare, both VCP-related FTD and ALS without ALS-FTD overlap or features of the IBMPFD spectrum have been described (110, 117). No specific genetic mutational signatures have been highlighted and the molecular mechanisms which distinguish one syndrome to the other are not clear (114).

Due to its highly variable phenotype, *VCP* disease is underdiagnosed and, whether present, genotype-phenotype correlations are not seen because of small sample size even in large cohorts. As awareness increases the scientific community is realizing that *VCP* disease is not as rare as previously considered. Hence, the experts suggest *VCP* molecular testing when two or more features are present for a prompt diagnosis, the adoption of surveillance protocols and hopefully the administration of a therapeutic option in the near future (114). As *VCP*-related phenotype has been expanding, IBMPFD term is currently misused in favor of the most inclusive and recently coined multisystem proteinopathy (MSP) (118). MSP is not restricted to *VCP* mutations, but other genes with similar functions have been shown to cause this severe disease (119).

For these reasons, it is even more crucial to understand the importance of VCP gene and its roles in the maintenance of homeostasis.

## The *VCP* Gene and Its Roles

**Figure 1:**
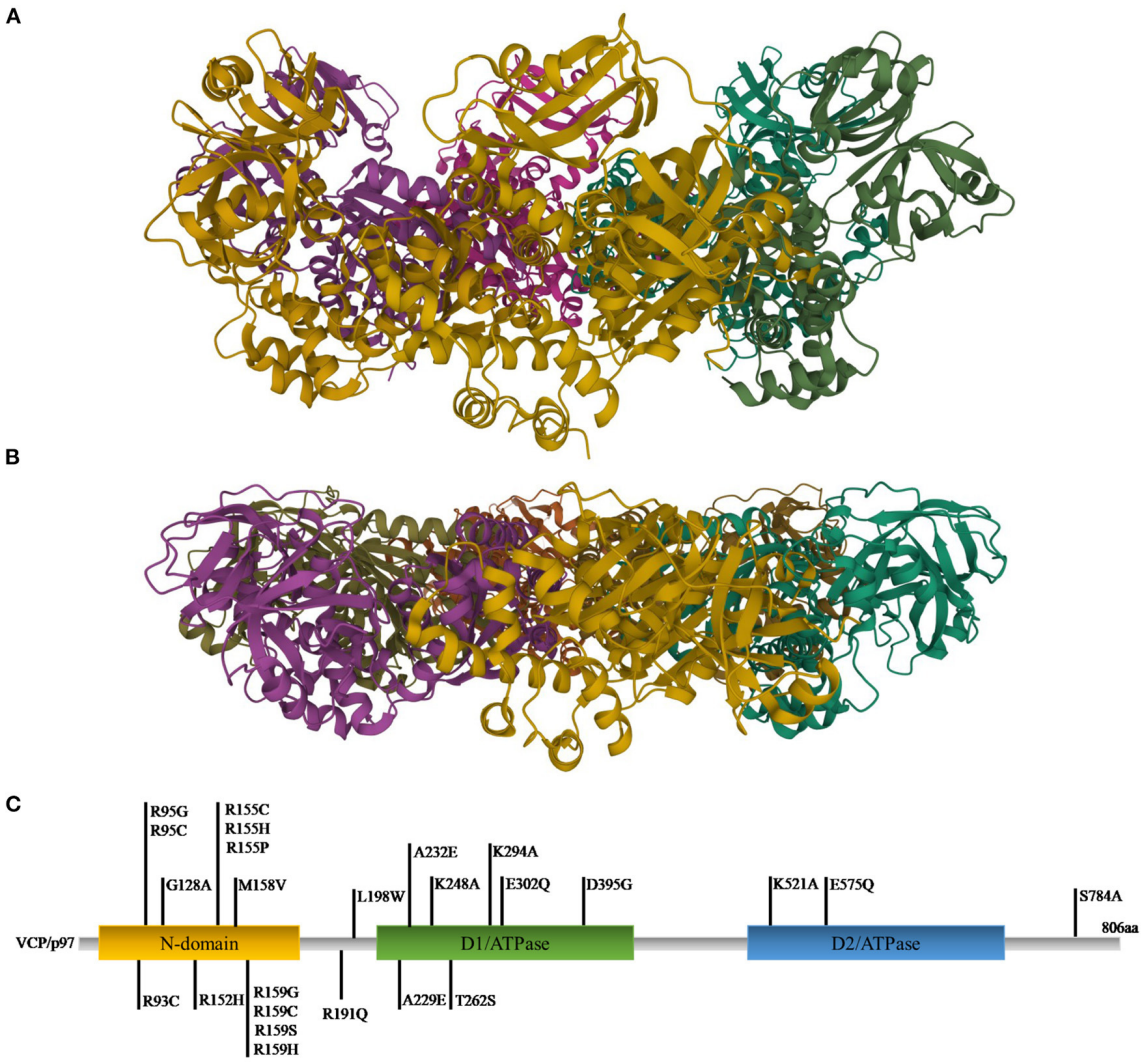
Ribbon diagram representations of X-ray structures of VCP in the up **(A)** and down **(B)** states [PDB ID code 4KO8 (122) for the up state and 1E32 (123) for the down state]. **(C)** Gene representation with the indication of the three main domains and the relative mutations (C-terminal domain was omitted).

VCP, also called p97, is a well-conserved protein among all eukaryotes (120, 121). It is encoded by the *VCP* gene, located on chromosome 9p13.3 in humans. VCP belongs to the type II AAA (ATPases Associated with diverse cellular activities) family and is composed of a N-terminal domain and a C-terminal domain, which interact with substrates and cofactors, and by two AAA ATPase domains, D1 and D2, forming a hexameric double-ring structures. D1 plays a role in hexamerization and regulates the heat-induced ATPase activity, whereas D2 acts better at physiological temperatures. Different studies showed that the N-terminal domain can assume two positions: above the plane of the ring (up position) (Figure 1A) or in the plane formed by the D1 ring (down position) (Figure 1B), with ATP or ADP in D1, respectively (124).

**Figure 2:**
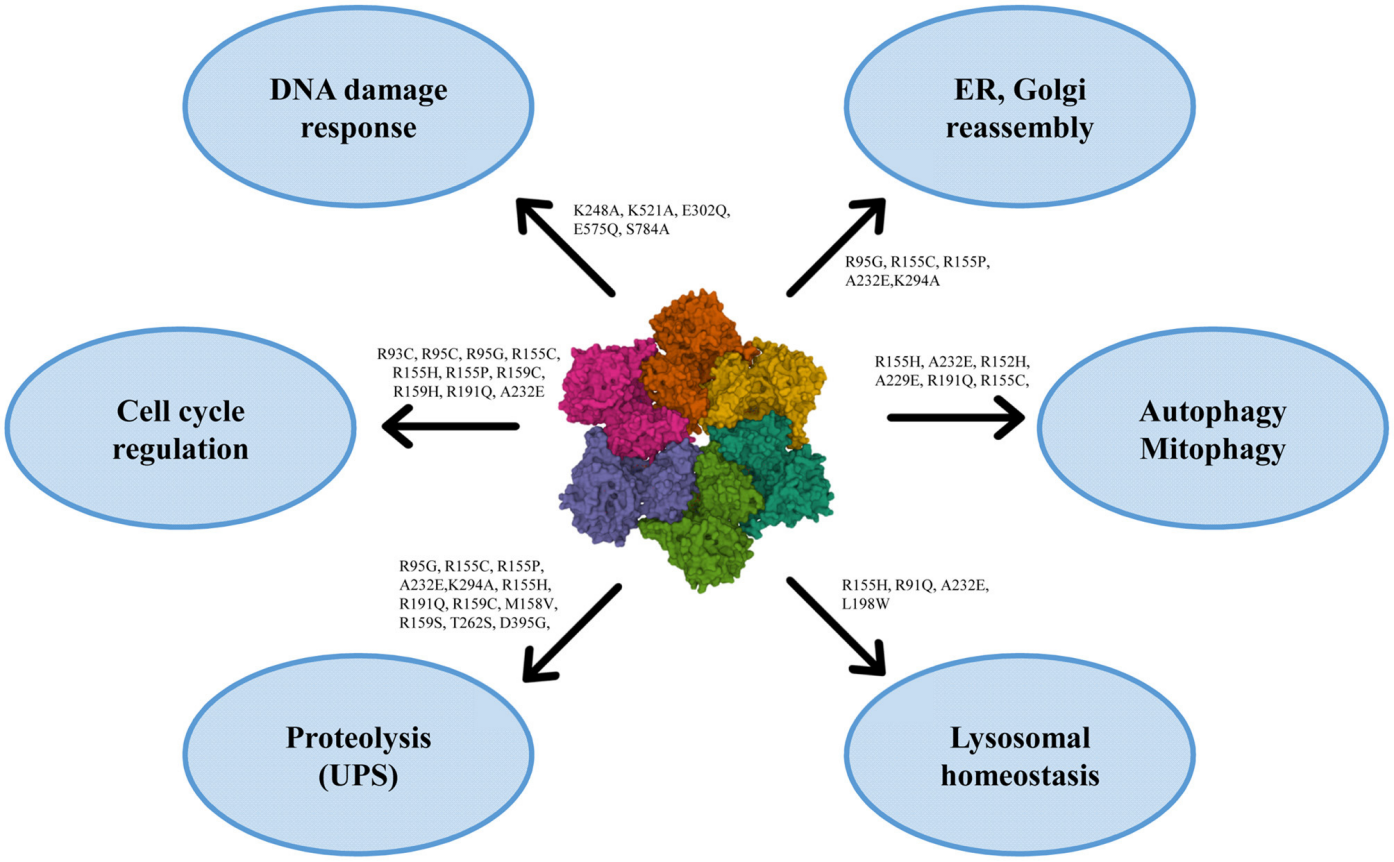
Roles of VCP in the cell with the principal mutations. VCP is involved in ER and Golgi reassembly, autophagy, mitophagy, lysosomal homeostasis, proteolysis, cell cycle regulation and DNA damage response [PDB ID code 3CF3 (126)].

VCP has multiple localizations: it is expressed in brain, skeletal muscles, ovary, testis, kidney, liver, heart, lung, lymph nodes, and whole blood, both in nucleus and cytoplasm (120, 121, 125). VCP uses the energy provided by the hydrolysis of ATP to change the conformations of target proteins, but it is also involved in different cellular processes, such as the reassembly of Golgi, endoplasmic reticulum (ER) and nuclear membranes, the ubiquitin-proteasome system, the regulation of cell cycle, the autophagosome maturation and the mitophagic process (121, 125) (Figure 2).

To exert its roles, VCP interacts with many cofactors, most of which bind the N-terminal domain, whereas a small number interacts with the C-terminal domain. Among the cofactors the most important are Npl4 (nuclear protein localization homolog 4), Ufd1 (ubiquitin fusion degradation 1) heterodimers and an arrangement of 13 UBX (ubiquitin regulatory X) domain cofactors. Mutations in *VCP* are causes of many pathologies (127) (Figure 1C). The first six mutations (R95G, R155H, R155P, R155C, R191Q, and A232E) were found in 2004 to be causative of the IBMPFD. This disease causes muscle weakness, cardiac problems, bone deformity but also amnesic and other cognitive deficits associated with TDP-43 inclusions (106, 128). Many studies revealed that the mutations are located in those domains important for cofactor binding and for VCP conformational changes (125).

In addition, mutations in *VCP* were found in other neurodegenerative diseases, such as Huntington Disease, ALS and FTD (125). In 2010 Johnson et al. identified the R191Q mutation in an Italian family affected by familial ALS. Moreover, further investigation in a wide range of ALS patients discovered four additional mutations, including the R155H one (110). More recently, a study on a cohort of 231 individuals with 15 different *VCP* mutations demonstrated that FTD was present in 30% of the patients, whereas 9% had an ALS phenotype, 4% had PD and 2% AD (116). More than 30 *VCP* mutations are till now reported in ALS and FTD and it is now accepted that in FTD they cause the unique subtype FTLD-TDP (type D), characterized by numerous neuronal intranuclear inclusions and dystrophic neurites in the neocortex (129, 130).

Mutations lead inevitably to an improper regulation of the VCP gene in exert its roles, among us in protein clearance, autophagy, lysosomal homeostasis maintenance and mitochondrial quality control.

## VCP and the Protein Clearance

One of the most important roles of VCP is its involvement in the clearance of proteins, especially in the ubiquitin-mediated protein degradation (125). In this process different cofactors are involved including the VCP–Ufd1–Npl4 complex which regulates different degradation processes, among which ERAD (endoplasmic reticulum-associated degradation), involved in the degradation of ER proteins and the maintaining of ER integrity. The VCP–Ufd1–Npl4 complex is also involved in the translocation of ubiquitinated ER proteins in the cytosol, where they are degraded by the proteasome, by the retro-traslocation (131, 132). With the same mechanism, the VCP–Vms1–Npl4 complex extracts the ubiquitinated substrates from mitochondria and transports them to the proteasome to be degraded (133, 134). Finally, VCP–p47 and VCP–UBXD1 complexes are involved in ubiquitinated protein degradation proteasome-independent; the two cofactors allow the transport of proteins to endosomes and the lysosomal degradation (125, 135). Moreover, it was demonstrated that Npl4 is required for normal microtubule and neuromuscular junction organization (136).

Mutations in *VCP* lead to a dysregulation of protein homeostasis with protein aggregation and accumulation, especially of TDP-43 and tau protein (134). Many studies demonstrated that down-regulation of *VCP* or mutations in this gene cause cytosolic TDP-43 aggregations followed by ER stress and cell death (137, 138). In 2010, Ritson et al. demonstrated that mutation in *VCP* causes TDP-43 redistribution in cytoplasm, both *in vitro* and in *Drosophila melanogaster*, gaining a cytotoxic function (139). Moreover, the knockdown of the cofactor Npl4 *in vivo* causes locomotor dysfunctions, reduced lifespan and inclusions of TDP-43 (136).

FTDL with ubiquitin-positive inclusions (FTDL-U) includes both sporadic and familial forms characterized by mutations in various genes, among which VCP. These inclusions are characterized by hyperphosphorylation, ubiquitination and fragmentation of the C-terminal (109, 140). Involvement of cortical and occipital lobes is more extensive in FTDL-U patients associated with *VCP*, and they were classified in the FTLD-TDP type 4, later renamed as type D (141). In 2018, a study on a cohort of FTD patients identified two different mutations, both characterized by the presence of diffuse TDP-43 immunoreactive neuronal intranuclear inclusions and dystrophic neurites and rare VCP inclusions (142), in contrast to a previous case report in which it was demonstrated the presence of VCP- and ubiquitin-positive cytoplasmic and nuclear aggregates in an Italian patients carrying the R159C mutation, and manifesting both body myopathy and FTD (143).

Inclusions of TDP-43 and its mislocalization in the cytoplasm was also observed in *VCP*-mutated iPSCs-derived MNs (144, 145) and in neuropathological examination of ALS patients (146, 147). TDP-43 was found accumulated also in cytoplasm of circulating lymphomonocytes as well in lymphocytes and monocytes separately evaluated in ALS patients (148).

A recent case study reported that the p.Asp395Gly mutation of *VCP* is associated in the occipital neocortex with aggregates immunoreactive with antibodies specific to phosphorylated tau and ubiquitin, characteristics similar to those of neurofibrillary tangles of AD (149). Moreover, the TDP-43 inclusions were often associated with FUS and splicing factor proline and glutamine rich (SFPQ) mislocalization, providing further evidence that *VCP* mutations cause an impairment of protein homeostasis (150). Often, the inclusions are associated with reactive microglia and astrocytosis and with cytokine imbalance (144, 149).

## VCP and Autophagy

Directly linked with the clearance of proteins is the role of VCP in autophagy. Autophagy is an essential process for all the eukaryotes cells. This process has two different purposes, the supply of amino acids for the cells in poor environmental conditions, the so called “adaptive autophagy”, and the degradation of proteins and damaged organelles, the “basal or constitutive autophagy” (151, 152). Many studies focused on the role of VCP in both the autophagic pathways, finding an involvement in both the types of process, cooperating with many different cofactors (127, 138, 153–156). First evidence highlighted the role of VCP exclusively in the maturation of phagosomes. In 2009 Ju et al. demonstrated that loss of the activity of *VCP* leads to autophagy impairment, causing the impossibility for vacuoles to mature in autolysosomes and the accumulation of autophagosomes. (138). These data were confirmed in 2010 by a study that found that knockdown of *VCP* or the overexpression of dominant-negative *VCP* (*VCP*^*R*155*H*^ and *VCP*^*A*232*E*^ mutations, involved in ALS/IBMPFD pathogenesis) lead to an accumulation of immature autophagic vesicles, often containing ubiquitin-positive contents and with acidified autophagosomes, defects found also when disease-associated *VCP* mutations were expressed (154). VCP regulates autophagosomes maturation in many different ways, such as interacting with clathrin and regulating caveolin trafficking or governing the size and the trafficking of endosomes regulating the assembly of EEA1 oligomers (135, 157, 158). VCP has been associated with the removal of damaged lysosomes and of stress granules (156, 159, 160). However, a recent study, using specific VCP inhibitors, discovered the role of VCP also in the first step of autophagy pathway, namely autophagy initiation. VCP can stabilize Beclin-1 acting on ataxin-3, which is responsible for Beclin-1 deubiquitination, and can also regulate the activity of the Beclin-1-containing phosphatidylinositol-3-kinase (PI3K) complex I which is responsible for the recruitment of downstream autophagy factors (161). Finally, a study using a *VCP*^*R*155*H*^ murine model, found a reduction in the mTOR targets EIF4EBP1 and RPS6KB1/p70S6 and as result an increasing autophagic activation and autophagosomes biogenesis (162).

Further studies focused on the *VCP* mutations typical of ALS and FTDL pathologies. In 2009 Ju et al. analyzed IBMPFD muscles and found degenerating fibers, rimmed vacuoles, which co-localized with autophagosomes-associated proteins such as Map1-LC3 (LC3) and p62/sequestosome, ubiquitin and TDP-43 inclusions. Moreover, they used U20S cells to silence *VCP* or to express an inactive VCP, confirming that it causes autophagosomes accumulation, which are unable to mature in autolysosomes, and to the accumulation of vacuoles and TDP-43 (138). Furthermore, a *VCP*^*R*155*H*/*R*155*H*^ homozygote mouse model showed growth retardation, weakness and pathological abnormalities of muscle fibers and brain. A histopathological analysis revealed TDP-43 positive sarcoplasmic inclusions, ubiquitin aggregates and a higher expression of the autophagosome marker LC3 when compared to wild type mice. In addition, p62 was detected in the cytoplasm and nuclear areas. These data were also confirmed in the brains of mutant mice; a histological analysis revealed an increase of LC3-I/II immunoreactivity and increased levels of TDP-43, ubiquitin aggregates and LC3-I/II proteins (163). More recently, Kustermann et al. demonstrated that loss of VCP *in vivo*, as can happen in ALS/FTD mutations, compromised protein degradation *via* autophagic pathway (164).

## VCP and Lysosomal Homeostasis

VCP has an important role in maintaining lysosomal homoeostasis. Lysosome dysfunction has been linked to many neurodegenerative diseases (165). Accumulation of lysosomes has been observed in muscle and myoblasts in IBMPFD/ALS patients carrying *VCP* mutations (138, 154). The endolysosomal pathway can be affected by membrane rupture and by lysosomal membrane permeabilization. Moreover, reactive oxygen species or protein aggregates can induce lysosomal damage (166). Damaged lysosomes are removed through the autophagic pathway in the so-called lysophagy process. The autophagosomal membrane associates the damaged organelle thanks to the ubiquitination of organelles components, which allows the interaction of the LC3-adapter proteins, such as p62/SQSTM1, and of the autophagy machinery (156).

In 2017 Papadopoulos and colleagues demonstrated the involvement of VCP in the endolysosomal damage response in Hela cells. They found that in cells depleted of *VCP* by siRNA, the clearance of damaged lysosomes was dramatically inhibited with an accumulation of the organelles. Moreover, *VCP* is essential for restoration after lysosomal damage, having regard to the fact that its silencing leads to cell death. The authors confirmed the data also in mice carrying the mutations typical of ALS and FTD, such as R155H and A232E. After the treatment to induce lysosomal damage, mouse embryonic fibroblasts showed an increase of LC3-II and an accumulation of lysosomes, suggesting an impairment of the lysophagic process. In the process UBXD1, PLAA, and YOD1 cofactors are importantly involved; they target specifically lysin 48-linked ubiquitin conjugates on lysin 63-decorated damaged lysosomes (156). The inactivation of VCP in skeletal muscle of adult mice leads to a necrotic myopathy, preceded by upregulation of LGALS3/Galectin-3, a typical marker of damaged lysosomes, and to the accumulation of autophagic proteins and of damaged lysosomes (167).

In 2019 Koerver et al. found that the ubiquitin-conjugating enzyme UBE2QL1 is essential for the coordinating of lysophagy process, associating with lysophagy effectors. They demonstrated that its knockdown abrogates the recruitment of VCP and consequently lysosomes clearance (168).

Moreover, a recent study defined the role of a specific VCP cofactor, the Small VCP-Interacting Protein (SVIP) in an animal model. It recruits VCP to lysosomes and its loss causes the disruption of the lysosomal network with an impairment of the autophagosome-lysosome fusion (169).

## VCP and Mitochondrial Quality Control

Finally, VCP is important in maintaining mitochondrial function and consequently calcium homeostasis. As previously explained, VCP is involved in the extraction of misfolded proteins from both ER and mitochondria and in their degradation. Moreover, it participates in the regulation of calcium homeostasis through mitochondria-associated ER membranes (MAMs) and in regulating mitochondrial calcium intake acting on the mitochondrial calcium uptake (MICU) proteins (134). Furthermore, experiments on *Drosophila* larvae revealed that VCP regulates the axonal transport of mitochondria directly interacting with Dynein (170); downregulation of *VCP* or its mutation decreased mitochondrial density in axons and increased their time of retrograde transport (170).

Numerous studies revealed signs of mitochondrial dysfunctions and aberrations in neurodegenerative diseases (134, 171). Damaged mitochondria have a depolarization of the inner membrane leading to their autophagic degradation called mitophagy. Mitophagy is mediated by the E3 ubiquitin ligase Parkin in a VCP-mediated manner. The inhibition of VCP prevents mitochondrial fusion and consequently mitochondrial elimination (155). These data were further confirmed in a study which demonstrated *in vivo* that VCP is required for the degradation of mitofusins and for the subsequent clearance of the damaged mitochondria. Moreover, the expression of A232E *VCP* mutation leads to a failure in mitochondria clearance and to a mitochondrial aggregation (172). Furthermore, in 2017 Zhang and colleagues demonstrated that the inhibition of *VCP* mutants *in vivo* suppresses mitochondrial defects, cell death and muscle damage (173). In this function VCP is assisted by different cofactors: UBXD1 translocates to mitochondria and promotes the VCP recruitment (174), whereas the cofactor UBXN1/SAKS1 facilitates Mfn2 degradation from mitochondria (175).

It was demonstrated that fibroblast, cortical neurons and motor neurons carrying the *VCP* mutations R191Q and R155C exhibited mitochondrial dysfunction and oxidative stress. Mitochondria had a decrease in membrane potential consistent with uncoupling of oxidative phosphorylation, as already found in fibroblasts from IBMPFD patients (176), and a higher rate of ROS production (144). The uncoupling results in a reduction in ATP production and consequently in a more vulnerable state of the cells (176, 177).

Finally, it was also demonstrated that *VCP*^*R*155*H*/*R*155*H*^ mice manifest striking mitochondrial abnormalities. Electron microscopy revealed abnormal mitochondrial structures with megaconia and disrupted cristae. Moreover, an increase in oxidative fibers and higher density of mitochondria were found out in muscle tissues (163).

## Therapeutic Approaches

Despite many studies focusing on neurodegenerative pathogenesis and the advances in the characterization of the involved pathways, there are no available therapies that are able to revert or halt the pathological process. However, some evidence highlighted possible therapeutic approaches for *VCP* mutation diseases, such as ALS and FTD.

In recent years, many different VCP inhibitors have been described (178). In 2017 Zhang et al. tested the inhibitors NMS-873 and ML240, drugs developed for the treatment of cancer, on an IBMPFD *Drosophila* model. They discovered that the administration of these drugs in wild type animals results in mitochondrial elongation, similar to what happens with *VCP* RNAi, and in a reversion of mitochondrial defects in wild type and mutant animals. These molecules prevent muscle cell death and restore the muscle integrity, the mitochondrial size, the structure of cristae and the myofibril organization in VCP mutants. Moreover, the treatment increases the levels of mitofusins and of the oxygen consumption rate of patient's fibroblasts (173). Later on, it was demonstrated that the treatment with ML240 can reverse the mislocalization of TDP-43, FUS and SFPQ in mutant motor neurons (131).

Another well-studied drug used for the treatment of *VCP*-related diseases is rapamycin. In cells with accumulation of TDP-43, the administration of 0.5 g/ml rapamycin reduces TDP-43 mislocalization acting on mTOR, a negative regulator of autophagy (179). Moreover, the LC3-II/LC3-I ratio was increased, revealing an increase in autophagosomes formation (179). Similar results were obtained in 2015 by treating *VCPR155H/*+ mice with 3 mg/kg body weight rapamycin, three times a week for 8 weeks. Treated mice showed improvement in muscle performance and autophagy markers, reduced levels of apoptosis and rescue of ubiquitin and TDP-43 pathology (180). However, further studies in *VCP*-RH mutant mice revealed that a chronic treatment with rapamycin can worsen the degenerative phenotype by decreasing muscle strength, and increasing vacuolated and atrophic fibers (162), as what happens after treatment with chloroquine (180).

Finally, in 2015 Nalbandian et al. excised the R155H mutation in a mouse model and developed Cre-ERTM-VCPR155H/+ tamoxifen-inducible mice. These recombinant animals improved muscle strength, autophagy pathway and decreased apoptosis (181).

## Conclusive Remarks

ALS and FTD have been considered for many years as two distinct pathologies with different pathological mechanisms. However, in recent years many studies have revealed that these diseases belong to a continuum, the so-called ALS-FTD spectrum. Several causative genes are shared in both diseases; one intriguing gene is ATPase *VCP*, already known for its relation to other muscle and neuropathic disorders, whose recent characterization and function determination has been shedding light into the dysfunctional molecular pathways in both ALS and FTD patients. In physiological conditions, VCP is involved in many different biological functions, namely protein degradation, autophagy, lysosomal and mitochondrial homeostasis, whose dysfunction hampers cell survival. Hence, its mutation can lead to severe or lethal consequences for the affected people. This review summarized the latest findings about the roles of VCP and about ALS-FTD mutations in this gene. Finally, we reported some evidence of possible therapeutic approaches targeting the *VCP* pathway.

## Author Contributions

ES and GF wrote the manuscript. IP generated figures. LD, OP, and SG helped write and reviewed the manuscript. All authors contributed to the article and approved the submitted version.

## Funding

This work was financially supported by the Italian Ministry of Health (Ricerca Corrente 2020-2021 and Ricerca Finalizzata Giovani Ricercatori 2016) and by Agenzia di Ricerca per la Sclerosi Laterale Amiotrofica (AriSLA) Foundation.

## Conflict of Interest

The authors declare that the research was conducted in the absence of any commercial or financial relationships that could be construed as a potential conflict of interest.

## Publisher's Note

All claims expressed in this article are solely those of the authors and do not necessarily represent those of their affiliated organizations, or those of the publisher, the editors and the reviewers. Any product that may be evaluated in this article, or claim that may be made by its manufacturer, is not guaranteed or endorsed by the publisher.

## Footnotes

^1^ https://alsod.ac.uk/output/gene.php/SOD1

^2^ https://alsod.ac.uk/output/gene.php/TARDBP

^3^ https://alsod.ac.uk/output/gene.php/FUS
